# A Comparison of Short-Term Outcomes of Robotic-Assisted Thoracic Surgery Versus Video-Assisted Thoracic Surgery Following Lung Cancer Surgery at a Tertiary Hospital in the United Kingdom: A Propensity-Matched Analysis

**DOI:** 10.7759/cureus.64298

**Published:** 2024-07-11

**Authors:** Nicolas W Mwesigwa, Vasileios Tentzeris

**Affiliations:** 1 Cardiothoracic Surgery, Hull University Teaching Hospitals NHS Trust, Hull, GBR; 2 Thoracic Surgery, Hull University Teaching Hospitals NHS Trust, Hull, GBR

**Keywords:** lung surgery, surgery of the lung, lung lobectomy, vats video assisted thoracoscopic surgery, robotic thoracic surgery

## Abstract

Background: Robot-assisted thoracic surgery (RATS) is gaining popularity in lung resection surgeries; however, its quality outcome measures require further evaluation. This study compared the short-term perioperative outcomes of lung resection surgeries performed using RATS and video-assisted thoracic surgery (VATS) at a tertiary hospital in the UK.

Methods: We performed a retrospective comparative analysis of 496 patients who underwent lung resection surgery at Castle Hill Hospital in the UK between January 2021 and April 2024. In the pre-matched cohort, 162 patients underwent RATS compared to 334 who underwent VATS. Using propensity matching based on the patient's forced expiratory volume in one second (FEV1) percentage of predicted age and body mass index (BMI), we included 324 patients in the analysis. Of these, 162 underwent RATS, and 162 underwent VATS, demonstrating satisfactory performance indicators.

Results: The results from our analysis depicted that RATS had a significantly lower rate of prolonged air leak (≥7 minutes) than VATS (5.5% versus 7.1%, mean difference -1.32, 95% CI: -0.89-3.08, p = 0.034). RATS patients also had shorter duration of hospital stay (3.8 ± 4.1 days versus 4.7 ± 4.8, mean difference -0.901, 95% CI: -1.886-0.084; p = 0.073) and had more mediastinal lymph node dissections (39.5% versus 35.2%) than VATS. However, the proportion of patients who were upstaged after histopathological analysis of the resected lymph nodes was not different between the two groups. Furthermore, the groups had no significant differences regarding the infection rate, intermittent positive pressure ventilation (IPPV) use, and theatre return.

Conclusion: Robotic and video-assisted technologies produced equivalent results for the majority of the short-term outcomes evaluated. Additional research is necessary to confirm RATS's efficacy and determine its potential advantages over VATS for lung resection surgeries.

## Introduction

To date, surgical resection remains the first-line treatment for patients with early-stage non-small-cell lung cancer (NSCLC) [1]. Surgeons initially performed lung cancer surgeries via thoracotomy, which required marked widening of the rib spaces. However, advancements in medical technology in the early 1990s introduced video-assisted thoracic surgery (VATS). Since then, VATS has increasingly replaced open surgery, becoming the preferred method for lung cancer resection [2]. VATS is majorly favoured due to decreased post-operative morbidity, reduced pain, reduced hospitalisation periods, improved physical function recovery, and oncological outcomes that are at least as good as, if not better, those of open thoracotomy [3-6].

Despite its enormous advantages, VATS still faces certain technical limitations, such as limited range of motion, the fulcrum effect, reduced dexterity, and the magnification of physiological tremors [7]. To overcome these limitations, robot-assisted thoracic surgery (RATS) has emerged as a new platform for lung cancer surgery. Between 2011 and 2015, the number of centres performing robot-assisted lobectomies surged by 90% globally. For instance, 320 RATS thoracic interventions in Germany alone were conducted in 2018 compared to just five in 2013 [8]. Similarly, the US jumped to 16.2% in 2013 from just 0.3% in 2009 [9]. This trend is widely expected to increase further with the advent of soft robotics and 3D and 4D folding instruments. Despite the rapid growth, large-scale adoption of RATS is still hindered by its exorbitant cost compared to VATS.

Available outcome data comparing VATS versus RATS for major lung resection surgery are limited and widespread, making interpretation and conclusion on which system is better challenging to ascertain. Current data from high-volume surgical centres suggest that both systems offer similar long-term oncological outcomes [10,11]. Therefore, quality outcome measures including perioperative complications, duration of hospital inpatient stay, and nodal upstaging will be crucial in helping surgeons decide on which approach to adopt. Findings from other smaller-scale surgical centres are also crucial in beefing up the available data, which forms the basis for decision-making. While new studies continue to emerge, their findings remain contradictory. For instance, while early reports on RATS lobectomy indicated higher rates of intraoperative injuries, bleeding, and longer operating times compared to VATS, subsequent reports have shown no significant difference in complication rates between the two systems [12,13]. This comparison is further complicated by the limited technical details reported in these studies and the surgeons at different RATS learning curve stages.

In this study, we retrospectively compared the clinical outcomes of RATS versus VATS for conducting lung resection surgery at a tertiary hospital in the UK. The results of this analysis will provide further evidence to assist surgeons and patients in deciding which approach to take.

## Materials and methods

Study design and patient selection

This study was a retrospective, comparative cohort audit and study involving adult patients who had lung cancer resection surgery using either RATS or VATS at Castle Hill Hospital in Yorkshire, England, from January 2021 to April 2024. The hospital audit department approved the study. Due to the study's retrospective design, informed patient consent was not required. The study included patients with a confirmed lung cancer diagnosis through histopathological analysis of resected specimens, regardless of resection extent or disease stage. Excluded were patients under 18 years of age and those who underwent lung resection for indications other than lung cancer.

Patient management

All preoperative patient evaluations were conducted following the clinical practice guidelines from the National Cancer Comprehensive Network [14], the American College of Chest Physicians [15], and the European Respiratory Society/European Society of Thoracic Surgeons (ESTS) [16]. Both groups (VATS and RATS) of patients received standardised post-surgery management as per the departmental protocol.

Operation technique

Robot-assisted procedures were conducted using the da Vinci Xi surgical system. This state-of-the-art robotic platform provides enhanced dexterity and precision, while VATS was performed using two or three ports depending on surgeon preference. Pulmonary vessels and bronchi were divided using automatic staplers per surgical preference and the operative situation encountered. Lymph node dissection generally included stations 2R, 4R, 7, 10R and 11 for right-sided tumours, and stations 5, 6, 7, 10L and 11 for left-sided tumours, with station 9 included for any lower lobe tumours. Peripheral parenchymal lesions lacking a previous tissue diagnosis underwent limited resection (wedge) with frozen section analysis. Anatomic resection was completed when cancer was confirmed histologically.

Variables and outcomes

We examined and analysed electronic medical records and the thoracic surgical department's database to collect data on various perioperative parameters, the surgical approach and the definitive procedures performed. The outcomes of interest included length of hospital stay, prolonged air leak (PAL), intermittent positive pressure ventilation (IPPV), infection and theatre return. Based on the eighth edition of the lung cancer stage TNM classification, staging was reported. A PAL is an air leak lasting more than seven days.

Statistical analysis

Statistical analyses were conducted using R version (4.2.2., The R Foundation for Statistical Computing, Vienna, Austria) and Statistical Package for the Social Sciences (IBM SPSS Statistics for Windows, IBM Corp., Version 24.0, Armonk, NY). Data were summarised using frequencies and percentages for categorical variables and means with standard deviations for continuous variables. Comparisons between groups were made using the t-test for continuous variables and Fisher's exact test or the Chi-squared test for categorical variables. To minimise selection bias due to non-random treatment allocation, patients were matched based on covariates considered factors associated with choosing RATS or VATS and the outcome of interest. The matching characteristics included forced expiratory volume in one second (FEV1) percentage of predicted age and BMI. The propensity score for matching was created using a logit model and was performed using the nearest neighbour approach, matching each RATS patient to one VATS patient. Covariate balance after matching was assessed with standardised differences and summaries of mean and median bias across all covariates before and after matching. Treatment effects comparing RATS and VATS were reported as mean differences for continuous outcomes and risk differences for categorical outcomes, with 95% confidence intervals and p-values. All p-values were two-sided, with statistical significance defined as p < 0.05.

## Results

The study included 496 patients. Of those, 162 (33.7%) underwent RATS, and 334 (67.3%) underwent VATS. This initial discrepancy is explained by VATS being well established by most thoracic surgical centres, where it has been the primary operation technique for the last 20 years.

RATS is still not widely available, mainly due to the financial implications required to initiate the service. The characteristics of the patients before propensity matching are detailed in Table 1.

**Table 1:** Patient characteristics before propensity score matching SD: standard deviation; BMI: body mass index; COPD: chronic obstructive pulmonary disease; FEV1: forced expiratory volume in one second; FVC: forced vital capacity Student’s t-test was conducted for the age variable, while Pearsons’s Chi-square or Fischer’s exact test was conducted for the other variables. P < 0.05 indicates statistical significance.

Variables	RATS (n = 162)	VATS (n = 334)	p-value
Age, years, mean ± SD	66.4 ± 10.2	62.5 ± 17.2	0.007
Sex, n (%)			
Male	74 (45.7)	182 (54.5)	
Female	88 (54.3)	152 (45.5)	
BMI (kg/m^2^) mean ± SD	28.7 ± 6.1	26.4 ± 5.6	0.000
Smoking status, n (%)			
Non-smoker	23 (14.2)	76 (22.7)	0.088
Smoker	19 (11.7)	57 (17.1)	
Ex-smoker	119 (73.5)	197 (59.0)	
Unknown	1 (0.6)	4 (1.2)	
Pulmonary function			
FEV1%, mean ± SD	79.1 ± 23.0	80.2 ± 25.3	0.642
FVC%, mean ± SD	95.8 ± 21.3	99.1 ± 21.4	0.183
Renal function, mean ± SD			
Creatinine (μmol/L)	77.3 ± 18.5	79.1 ± 30.6	0.496
Urea (mmol/L)	4.2 ± 0.3	5.5 ± 2.6	0.000
Comorbidities, n (%)			
Insulin-dependent diabetes	8 (4.9)	6 (1.8)	0.078
Ischaemic heart disease	18 (11.1)	32 (9.6)	0.634
Cardiac failure	2 (1.2)	7 (2.1)	0.725
Previous stroke	15 (9.2)	26 (7.8)	0.604
Steroid therapy	7 (4.3)	14 (4.2)	1.000
Anticoagulation	32 (14.1)	50 (14.9)	0.198
Previous history of cancer	60 (37.0)	99 (29.6)	0.102
Hypertension	53 (32.7)	130 (8.9)	0.198
Alcoholism	10 (6.2)	15 (4.4)	0.512
Hyperlipidaemia	76 (46.9)	114 (34.1)	0.006
Peripheral vascular disease	14 (8.6)	21 (6.3)	0.353
Induction chemotherapy, n (%)	13 (8.0)	29 (8.6)	0.865
Induction Radiotherapy, n (%)	16 (9.8)	37 (11.0)	0.758
Histopathology, n (%)			
Squamous cell carcinoma	25 (15.4)	21 (6.2)	0.071
Adenocarcinoma	76 (46.9)	97 (29.0)	
Carcinoid tumors	7 (4.3)	5 (1.5)	
Others	4 (2.5)	2 (0.6)	

Patients who underwent RATS were significantly older (p < 0.05) and had higher BMI (p < 0.001) than VATS patients. Of the performed procedures, 200 (40.3%) were lobectomies, 56 (11.3%) were bi-lobectomies, 224 (45.2%) were wedge resections and 16 (3.2%) were procedures other than the above. The type of procedure differed significantly between the two groups. By type of procedure, more lobectomies were performed with VATS compared to RATS (64% vs 36%, p < 0.05). Similarly, more wedge resections were conducted with VATS than RATS (72.3% vs 27.7%, p < 0.05).

Following propensity score matching, 324 patients were selected and analysed. Of those, 162 underwent RATS and 162 VATS. Prior to propensity matching, patient characteristics were grossly similar; however, differences were observed mainly with age and BMI. These were corrected with matching and performance indicators were deemed satisfactory. Table 2 displays the matching characteristics of the groups.

**Table 2:** Matching characteristics of patients BMI: body mass index; VATS: video-assisted thoracic surgery; RATS: robotic-assisted thoracic surgery; FEV1: forced expiratory volume in one second

Variable	All patients		Propensity-matched patients	
	RATS	VATS	RATS	VATS
FEV1 % of predicted, mean	79.14	80.23	79.14	80.72
Age in years, mean	66.44	62.50	66.44	66.93
BMI in kg/m^2^, mean	28.72	26.38	28.72	28.13

The postoperative outcomes are presented in Table 3.

**Table 3:** Surgical outcomes after propensity score matching VATS: video-assisted thoracic surgery; RATS: robot-assisted thoracic surgery; SD: standard deviation; IPPV: intermittent positive pressure ventilation; CI: confidence interval; TNM: tumour, node, metastasis A prolonged air leak is an air leak lasting longer than seven days after the operation. Length of hospital stay was measured in the days with their mean ± standard deviations. In contrast, prolonged air leak, IPPV, infection rate, theatre return, mediastinal node dissection and TNM staging were measured in percentages. Student’s t-test was conducted for the length of hospital stay variable, while Pearsons’s Chi-square or Fischer’s exact test was conducted for the other variables.

Outcome	RATS (n = 162)	VATS (n = 162)	Difference between RATS vs. VATS (95% CI)	p-value
Length of hospital stay, days, mean ± SD	3.8 ± 4.1	4.7 ± 4.8	-0.901 (-1.886 to 0.084)	0.073
Prolonged air leak (%)	9 (5.5%)	12 (7.1%)	-1.32 (-0.89 to 3.08)	0.034
IPPV (%)	1 (0.6%)	0 (0.0%)	0.0 (-2.11 to 2.11)	0.317
Infection rate (%)	17 (10.5%)	10 (6.2%)	2.03 (0.7 to 5.19)	0.148
Theatre return (%)	1 (0.6%)	1 (0.6%)	0.00 (-4.07 to 4.07)	1.000
Mediastinal node dissection %	64 (39.5%)	57 (35.2%)	0.04 (-0.2 to 0.3)	0.565
TNM upstaging %				
cN0 to pN1	2 (1.2%)	3 (1.8%)	0.51 (-1.97 to 1.62)	0.652
cN1 to pN2	0 (0.0%)	1 (0.6%)	-0.0024 (-2.16 to 2.15)	0.317
T1b to T2a	5 (3.1%)	6 (3.7%)	-0.04 (-0.17 to 0.81)	0.759
T1b to T3	0 (0.0%)	2 (1.2%)	-0.17 (-1.44 to 0.83)	0.156
T2a to T3	0 (0.0%)	2 (1.2%)	-0.17 (-1.44 to 0.83)	0.156

The average length of hospital stay was slightly higher in the VATS group than the RATS group (4.7 ± 4.8 vs 3.8 ± 4.1; mean difference of 0.901; 95% CI: 0.084-1.886; p = 0.073) but was not statistically significant. There were no appreciable variations between the groups in terms of IPPV, post-operative infection, and return to theatre (all p > 0.05). PAL was significantly higher in the VATS group (7.1% vs 5.5%, risk difference of 1.32, 95% CI: 0.89-3.08, p = 0.034). More mediastinal node dissections were conducted with RATS than VATS (39.5% vs 35.2%, p = 0.565), however, upstaging of tumours following analysis of the resected lymph nodes was not significantly different between the two groups.

## Discussion

This study assessed the short-term clinical outcomes of VATS, an established method for lung surgical resection in most surgical centres, with RATS, a more recently validated alternative. While the intended goals of lung surgical resection, especially for lung cancer treatment, are to achieve thorough resection of the disease, reduce perioperative complications and enable quicker recovery, the preferred approach to achieve this continues to be debated.

In our propensity-matched cohorts, there was no significant difference between RATS and VATS for most short-term outcomes of interest. For instance, there was no difference in length of hospital stay, IPPV, infection rate, theatre return and pathological lymph node upstaging despite more mediastinal node dissection in the RATS group. However, a notable difference was seen in PAL, with more VATS cases than RATS (7.1% vs 5.5%, p = 0.034).

In this study, PAL is defined as a chest tube air leak persisting for seven days or more post-surgery. Its presence correlates with increased morbidity, characterized by prolonged hospital stays and chest tube placement, higher incidences of respiratory complications and pleural empyema, as well as elevated rates of reoperation, readmission, and in-hospital mortality [17,18]. Our findings are consistent with those of Farivar et al. [19] and Kneuertz et al. [20], who found reduced PAL rates following RATS lobectomy compared to VATS, as well as Muriana et al., who compared rates PAL rates with open thoracotomy [21]. Generally, VATS has been noted to perform inferiorly when faced with surgeries requiring incomplete lung resections, especially in complex segmentectomies requiring deeper, peripheral dissection of the segmental hilum [22]. Hence, a significant limitation of the technique is the possibility of achieving adequate surgical margins and lymph node dissection while providing precise dissection to prevent PAL onset [23]. Similarly, patients with incomplete or absent fissures are more challenging to operate using VATS [24]. To this effect, robotic platforms offer a better alternative, allowing for precise dissection of segmental structures and the use of dedicated staplers that ease deep tissue manoeuvres, significantly reducing PAL occurrence.

Our study further revealed that RATS procedures facilitated more lymph node dissections than VATS (39.5% vs 35.2%), although the difference was not statistically significant. This, however, did not translate into significant pathologic TNM upstaging similar to those of Lampridis et al. [25]. This result is also consistent with that of a recent meta-analysis of eight studies comparing RATS (n = 997) and VATS (n = 1144) for anatomic lung resection that found that RATS was associated with a higher number of dissected nodal stations (weighted mean difference, 0.51; 95% CI: 0.15-0.86; p = 0.005; I2 = 86%) [26]. This is attributed to RATS's unique ability to dissect deep tissues in complex anatomical locations, such as the mediastinum and pulmonary hila, which has been corroborated by multiple studies showing higher rates of pathologic lymph node upstaging with RATS. Similarly, a propensity-score-adjusted comparison of RATS vs VATS in pathologic nodal upstaging of 911 patients showed that RATS was superior (16.2% versus 12.3%; p = 0.03) [27]. Although our data did not show increased TNM upstaging in RATS, its ability to facilitate enhanced hilar and mediastinal lymph node dissection enables their thorough assessment, hence detection of occult metastases and, thus, more accurate staging of lung cancer.

Overall, the RATS group of patients had a comparable morbidity rate to the VATS group. However, RATS had superiority in a reduced rate of PAL and enhanced mediastinal node dissections. These findings further support the development of risk score assessment tools that aid the selection of RATS or VATS based on the objective maximum benefit to the patients. For instance, the ESTS and the Society of Thoracic Surgeons (STS) have both independently developed risk-scoring systems to stratify the risk of PAL onset following lung resection based on several preoperative characteristics [28,29]. Such a system could incorporate RATS as the recommended approach for lung resection based on more robust outcome data on its ability to minimise PAL.

Robotic surgery is relatively new in our department, with a few surgeons at the beginning of their learning curve. Outcomes can only improve with further experience gained. In addition, it was introduced just a year prior to the COVID-19 pandemic, which significantly disrupted patient flow, resulting in the few numbers observed in this study. As numbers come back to pre-pandemic levels, so will the number of surgeries and improved surgeon efficiency.

While interpreting this study, the following limitations should be taken into consideration:

1. Although patient matching was employed to reduce selection bias, the study's retrospective design may have influenced the outcomes.

2. Matching was only performed for a few clinically relevant characteristics at the time of surgery. Another potential bias stems from the selection of the surgical approach based on the surgeon's expertise and the patient's preferences.

3. Due to data limitations, we did not compare other key pre- and perioperative factors that are important in clinical decision-making.

Zhao et al.'s study [24] compares these factors. Similarly, we did not assess the average hospital costs per patient between the two approaches, which is a crucial factor in clinical decision-making amid rising healthcare expenses. A comprehensive cost comparison between the two approaches can be found in Harrison et al.'s study [30].

## Conclusions

In a propensity-matched analysis of lung resection surgeries, RATS and VATS showed similar results for most short-term outcomes. However, notable differences were observed in the rate of PAL after surgery and mediastinal node dissection. These discrepancies deserve cautious interpretation, as they may be influenced by the learning curve associated with RATS. As the use of RATS for lung cancer increases and more robotic platforms become available, ongoing research is essential to validate the integration of this technology and determine its potential advantages over VATS for broader use.
